# Supplementary material for: Imaging electric field dynamics with graphene optoelectronics
Source: Nat Commun. 2016 Dec 16;7:13704. doi: 10.1038/ncomms13704 (PMC5172231; doi:10.1038/ncomms13704)
Supplement: Supplementary Information — Supplementary Figures, Supplementary Notes and Supplementary References. [file ncomms13704-s1.pdf]

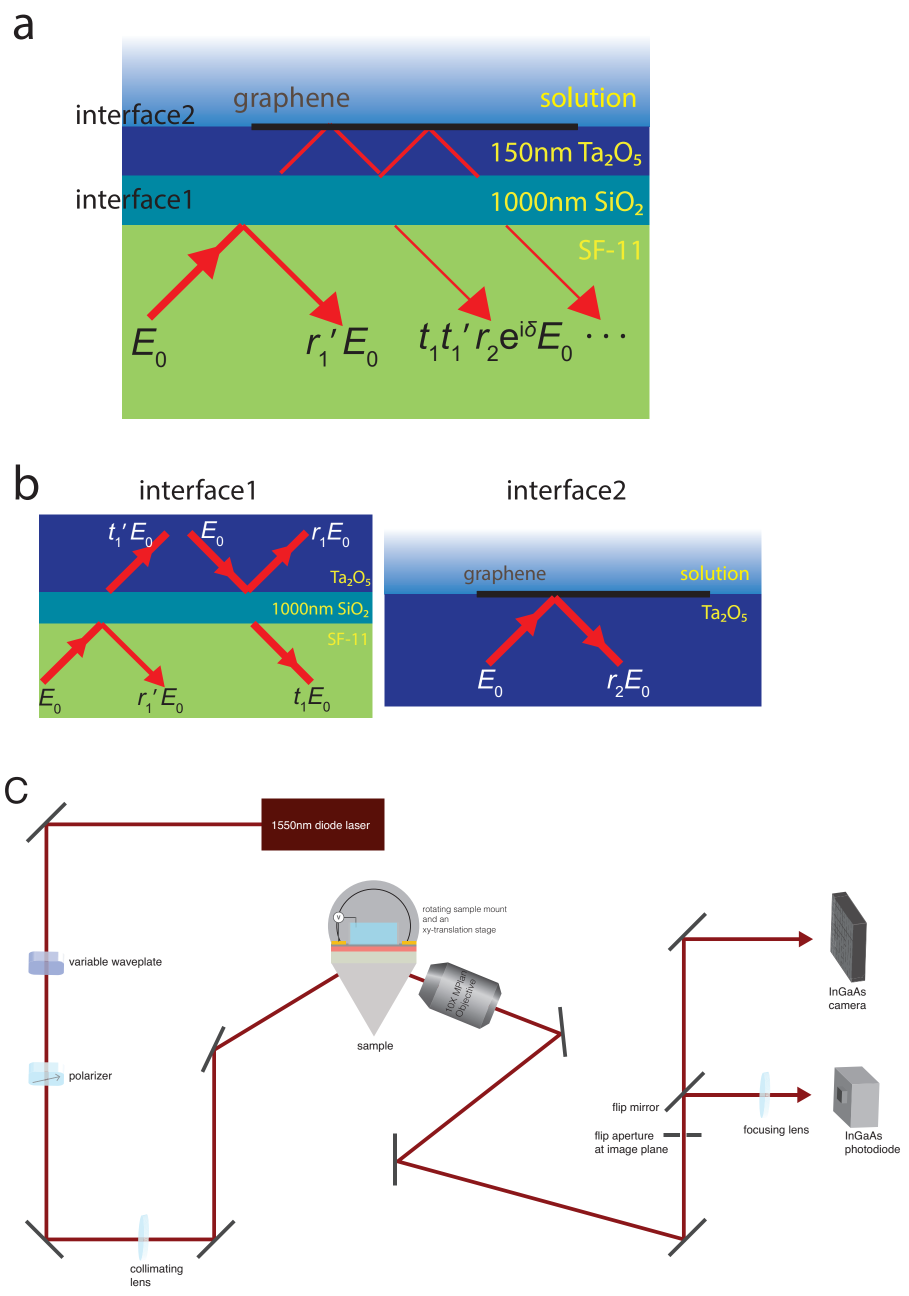

Supplementary Figure 1| Detailed analysis on the critically coupled waveguide structure. a, The geometry of the layered structure are shown (not in scale) with the multiple reflection analysis.Interface1 and interface2 are highly reflective and form a Fabry-Perot cavity. The definition of r_1',r_2 ,t_1,t_1' are given in (**b**) and in the text. **b**, Two sub-systems considered in S1: interface1 consists SF11/SiO_2(1000 nm)/Ta_2O_5 and interface2 consists Ta_2O_5/Graphene/water. Both of them form a highly reflecting mirror. **c**, Schematic of optical setup.

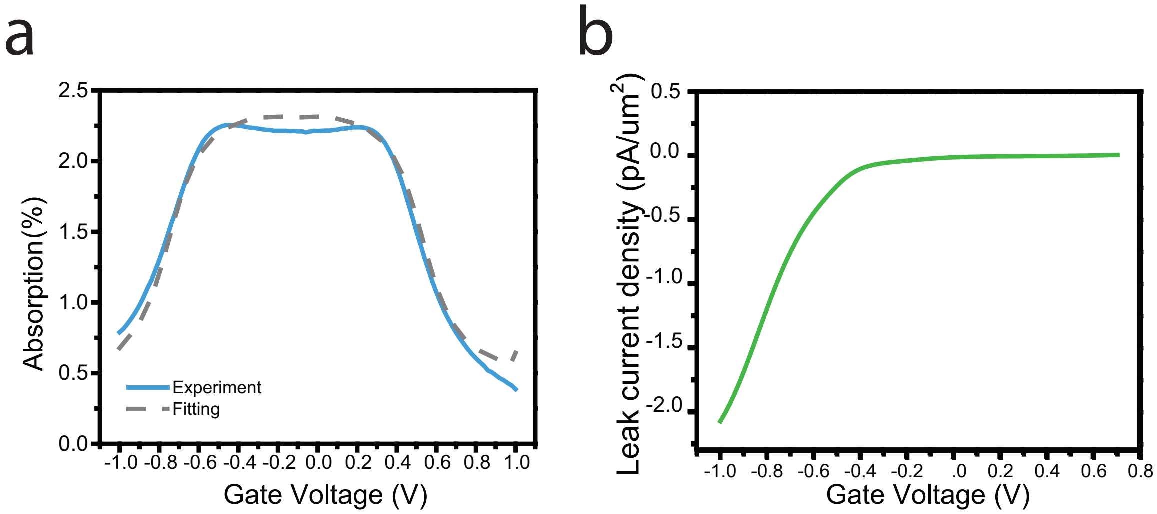

Supplementary Figure 2 | Gate dependence of optical absorption of monolayer graphene. a, Optical absorption measurement with a monolayer graphene on glass substrate at 1.55μm at normal incidence as a function of gate voltage via Ag/AgCl electrode through saline solution. **b,** Simultaneous current density measurement on graphene electrodes while during voltage scan in Supplementary Figure 2a. The current density is very small within the window of ±1V, indicating negligible chemical reaction between graphene and PBS solution.

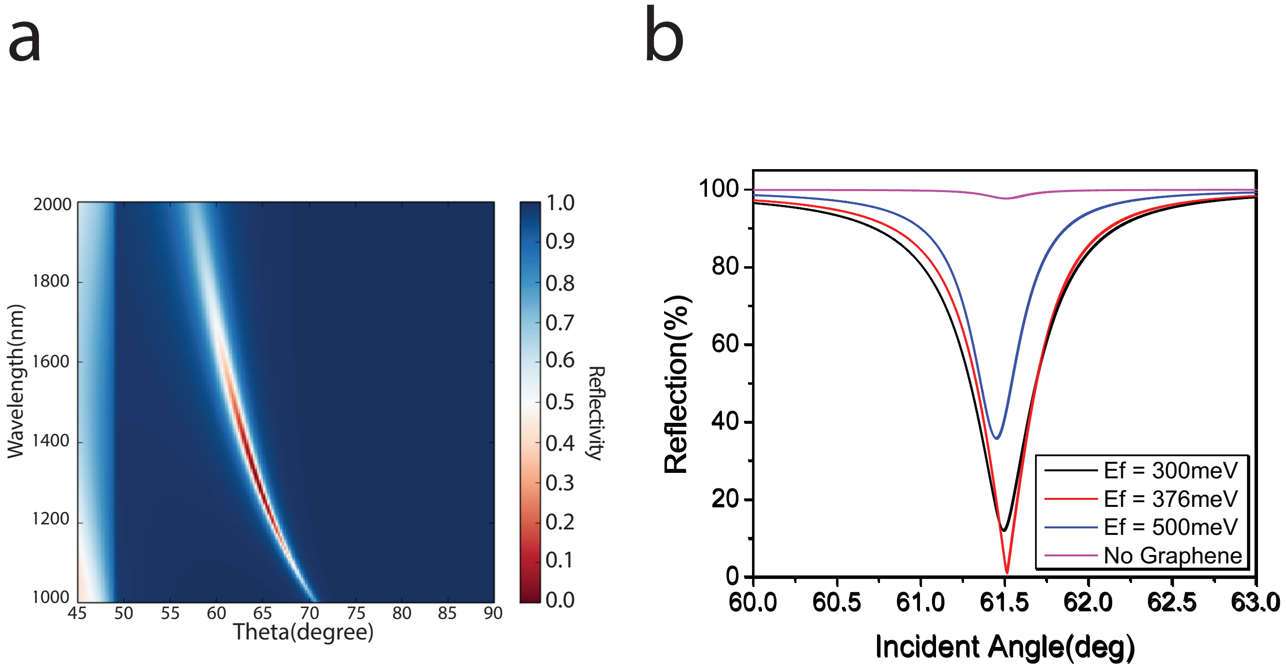

Supplementary Figure 3 | Simulation of optical response from the graphene optoelectronic electric field imaging device. a, Reflectance map of s-polarized planewaves with varying wavelengths and incident angles from the device structure illustrated in Figure 1b in the main text. Reflectance is reduced due to absorption of graphene only when the incident angle matches with 0th TE mode of the waveguide. **b,** Reflectance as a function of incident angles for different graphene Fermi energy. The depth of resonance dip can be controlled by tuning graphene's Fermi energy via an external gate.

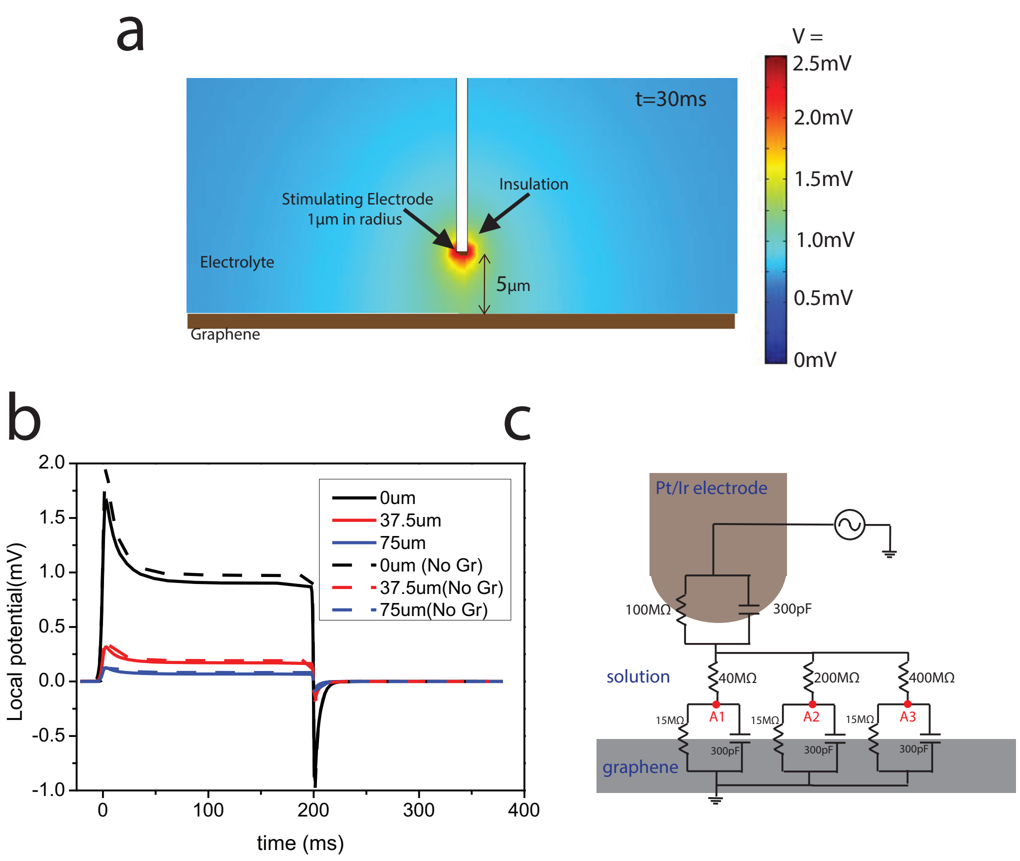

Supplementary Figure 4 | Finite-element simulation for microelectrode voltage modulation. a, Cross-section of simulation geometry and simulated potential distribution in solution at t = 30 ms. **b,** Dynamics of local potential (solid) in electrolyte just above graphene for positions corresponding to A1(below the tip), A2(37.5µm away) and A3(75 µm away). The same quantity without graphene is also shown for comparison as a grey dashed line. The simulation gives a qualitative description for the spatio-temporal phenomenon seen in experiment and indicates that our device acts a nonperturbative local field detector. **c,** An equivalent circuit may be used to describe the spatio-temporal dynamics in our experimental configuration.

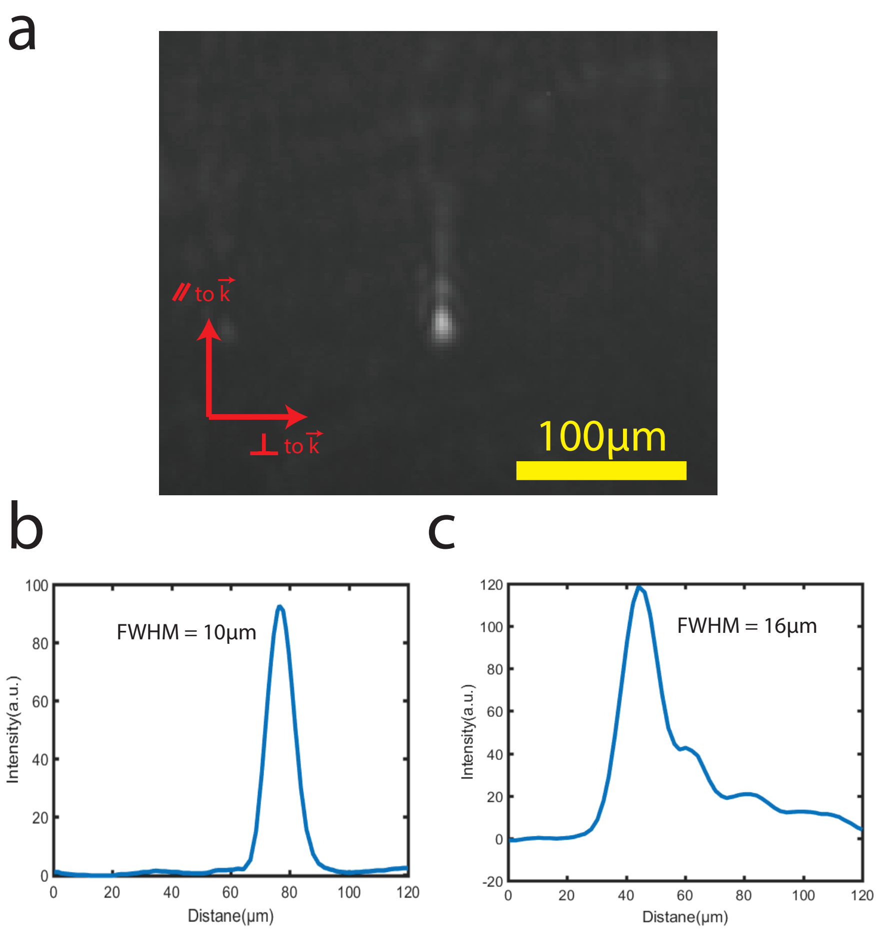

Supplementary Figure 5 | Calibration of spatial resolution of CAGE imaging system. **a,** Image of one microsphere with 1 µm diameter taken with CAGE optical system. **b,** Intensity profile on a line cut across the direction perpendicular to the light propagation direction ($\vec{k}$). Spatial resolution in this direction is 10 µm. **c,** Intensity profile on a line cut across the direction parallel to $\vec{k}$. The spatial resolution in this direction is 16 µm.

Supplementary Note 1: Description of critically coupled waveguide

Supplementary figure 1a shows a detailed analysis of the waveguide structure and describes the working principle of the device. To describe the interaction of the light with the multilayer device, we can separate the system into two sub-systems: one consists of SF11/SiO_2(1000 nm)/Ta_2O_5 and other consists Ta_2O_5/Graphene/solution as each of them form a highly reflecting surface. The sub-systems are shown in supplementary figure 1b. Supplementary figure 1c outlines the optical imaging setup.

The SF11/SiO_2(1000 nm)/Ta_2O_5 sub-system can be treated by standard methods of frustrated total internal reflection(FTIR). Here, we assume the reflection and transmission coefficients for light incident from the SF11 side are r_1' and t_1', while the coefficients from the Ta_2O_5 side are r_1 and t_1. One can prove that $|r_1| = |r_1'|$ and $t_1 t_1' = (1 - |r_1|^2)\exp{(\delta_1 + \delta_1' + \pi)}$[1], where δ_1 and δ_1' are the phase of $r_1$and r_1', respectively.

In the Ta_2O_5/Graphene/water sub-system, r_2 is defined as the reflection coefficient for light incident from the Ta_2O_5 side of the interface. r_2 can be calculated with the Fresnel equation and perturbation theory on graphene absorption

$$r_2 = 1 - A_{\mathrm{gr}} \times Re\left[\frac{(1 + r_2^0)^2}{r_2^0}\right] = 1 - A_{\mathrm{gr}} \times 1.66$$

where A_{gr} is the absorption of graphene, and r_2^0 is reflection coefficient at interface of Ta_2O_5/water without graphene.

We can now consider the optical properties of the combined system. As these two sub-systems are highly reflective, the combined structure consists of two parallel highly reflected mirrors and may therefore be described as a Fabry-Perot cavity. The total reflected radiation E_{r} is the interference of the multiple reflections and has the form:

$$\frac{E_r}{E_0} = r_1' + t_1 t_1' r_2 e^{i\delta}\left[1 + \left(r_1 r_2 e^{i\delta}\right) + \left(r_1 r_2 e^{i\delta}\right)^2 + \cdots\right]$$

$$= r_1' + \frac{t_1 t_1' r_2 e^{i\delta}}{1 - r_1 r_2 e^{i\delta}} \qquad \text{(Supplementary Equation 1)}$$

where $E_0$is the incident electric field, $e^{i\delta}$ the phase accumulationas the light propagates through the Ta_2O_5 medium. Given the relationship between r_1,r_1', t_1,t_1', supplementary equation 1 may be simplified to

$$\frac{E_r}{E_0} = e^{i\delta'}\frac{|r_1| - |r_2|e^{i\Delta}}{1 - |r_1||r_2|e^{i\Delta}} \qquad \text{(Supplementary Equation 2)}$$

where $\Delta = \delta + \delta_1 + \delta_2$ is the round trip phase accumulation in the waveguide. The resonance of the Fabry-Perot cavity takes place at $e^{i\Delta} = 1$, which is the same requirement for the zeroth mode for Ta_2O_5 planar waveguide.[2] The phase accumulation, Δ, depends sensitively on the incident angle, allowing one to find the resonance of the Fabry-Perot cavity by varying the incident angle coupling into the waveguide structure. By squaring the supplementary equation 2, one can get the reflectance formula shown in Eq1 in the text and the critical condition $|r_1| = |r_2|$, at which the reflection intensity dramatically decreases as the light has near-100% absorption into graphene (see main text for further discussion). Operating the device at the critical coupling condition can enhance the contrast significantly due to the strongly enhanced light-matter interaction and the low reflection background.

Supplementary Note 2: Optical absorption of monolayer graphene

To optimize the optical response of the CAGE device, we first examined the gate dependence of graphene's optical absorption in an ionic solution (see methods). We obtained an optical reflection measurement from monolayer graphene on glass substrate at 1.55 μm with normal incidence as a function of gate voltage through solution with a Ag/AgCl electrode. The absorption is derived from the reflection data based on Fresnel equations and shown in supplementary figure 2a. The absorption at zero-gate voltage is measured to be 2.19%, which is close to the universal optical absorption of graphene(2.3% theoretically). The absorption starts to decrease at around ±0.4V due to the band-filling effect and continues to decrease to ±1.0V. The absorption may be tuned from 0.47% to 2.19% over a gate voltage of ±1.0V.

The optical absorption of graphene, A_{gr}, contains contributions from both interband and intraband transitions in graphene. Its frequency dependence at different Fermi energies (E_F) can be approximated by[3,5]

$$A_{gr}(E_F) = \frac{\pi e^2}{\hbar c}\left[1 + \frac{1}{\pi}\left(tan^{-1}\frac{E-2|E_F|}{\Gamma} - tan^{-1}\frac{E+2|E_F|}{\Gamma}\right)\right] + \frac{4e^2}{\hbar c\tau}\frac{|E_F|}{E^2+(1/\tau)^2}$$

(Supplementary Equation 3)

where E is the incident photon energy, Γ, the interband and $1/\tau$ the intraband transition broadening, respectively. The Fermi level E_F varies with the carrier concentration, n, as $E_F = hv_F\sqrt{\pi n}$, where the Fermi velocity v_F is 1.1×10^6 m s^{-1}. In the electrolytic cell, the averaged carrier concentration,n_0, can be described by a capacitor model $n_0 = C(V_g - V_{CNP})/e$, where C is the double layer capacitance of the solution under Ag/AgCl gating, and V_{CNP}, the charge neutral point, is fitted to be -0.11V.

To account for the doping inhomogeneity in graphene, we introduce a local carrier concentration broadening of $\Delta = 5\times10^{11}\text{cm}^{-2}$. As a result, the conductivity of graphene can be described as

$$A_{gr}(n_0) = \frac{\int A_{gr}(n)e^{-(n-n_0)^2/\Delta^2}dn}{\int e^{-(n-n_0)^2/\Delta^2}dn} \qquad \text{(Supplementary Equation 4)}$$

The fitting results for the graphene absorption as a function of V_g are plotted in supplementary figure 2a (dashed line), where the fitted parameters both interband and

intraband broadening are 80meV, double layer capacitance $C = 2.07\ \mu F\ cm^{-2}$. From the absorption curve of graphene, we estimate that the voltage sensitivity in a transmission configuration $(dT/T)/dV$ = 0.0048% per mV due to intrinsic broadening of graphene interband transitions. The current density on graphene electrodes is monitored during the optical measurement and shown in supplementary figure 2b. The current is small within the window of ±1Vand the band-filling effect can be reproduced many times indicating a lack of a chemical reaction between graphene and the solution.

Supplementary Note 3: Simulation of optical response from device

To optimize the device structure, we built a custom Python simulation that explored materials, dimensions, tolerances, and coupling conditions to provide theoretical bounds on spatial resolution and field sensitivity. The simulation is based on transfer-matrix formalism. These simulated conditions included polarization and coupling angle of the infrared beam, graphene's optical properties in aqueous solutions, and the structure of planar waveguide layers. Drawing upon these results we found the optimal parameters, compatible with standard fabrication techniques, for the spatial and charge sensitivity. Supplementary figure 3a shows the reflection map of s-polarized planewaves with various wavelength of light and incident angles from a graphene-coated waveguide structure shown in Fig. 1b. in the main text. Most of the wavelength and incident angle combinations give unity reflection due to total internal reflection. Only when the incident angle matches the zeroth transverse electric mode(TE mode), can light couple into the waveguide and absorption by graphene becomes significant.

Supplementary figure 3b shows the reflection of s-polarized planewaves at 1.55 μm from the same waveguide structure as a function of incident angles for different graphene Fermi energies. The dips in the reflection curves are due to the zeroth transverse electric mode of the waveguide and the depth of resonance peak changes significantly for different Fermi energies of graphene. The optical absorption of graphene decreases monotonically with the Fermi energy and determines the fraction of light being absorbed in the waveguide. The simulation indicates that the strongest (deepest) resonance takes place at one specific Fermi energy (376 meV in supplementary figure 3b). At this Fermi energy, the absorption of graphene is such that the multiple reflections in the waveguide destructively interfere, resulting in a near-zero reflection of the probe beam.

Finally, we simulate optical response in a real experimental setting where the incident angle is fixed at the resonance condition and assuming numerical aperture N.A. = 0.002 by averaging the reflectance within the angle divergence(~0.6º). The absorption of graphene used in the simulation is taken from the experimental data from supplementary figure 2a. The simulated voltage-dependent reflectance and sensitivity of

the device is shown in Figure 2a and 2b in the main text as dashed lines, respectively, and may be compared with the experimental responses from the CAGE optical system.

Supplementary Note 4: Finite-element simulation of the microelectrode voltage modulation

To understand the spatio-temporal behavior of local potential measured in micro-electrode stimulation experiment shown in Fig. 4a in the main text, we use the Electrochemistry Module in COMSOL Multiphysics to simulate the electric potential in solution during applied voltage pulses. The simulation accounts the effect of the electrode kinetics in addition to solution resistance. The current distribution in electrolyte is assumed to obey Ohm's law and the electrode interface are simulated by a double-layer capacitance together with a linearized Butler–Volmer equation to describe electrode current, namely:

$$i_{el} = i_0 \frac{\alpha F}{RT} \eta \qquad \text{(Supplementary Equation 5)}$$

where i_{el} is the electrode current, i_0 the exchange current density, α the cathodic and anodic charge transfer coefficient, F the faraday constant, R the gas constant, T the temperature and η the overpotential of the electrode.

In the simulation, we use a 1µm-radius-disk stimulating electrode with $C = 10\text{F m}^{-2}$, $i_0 = 20\text{A m}^{-2}$, $\alpha = 1$ embedded in an electrolyte with conductivity $\sigma = 0.0022\text{ S m}^{-1}$.The parameters for stimulating electrode are provided by the manufacturer and electrolyte conductivity is from literature.[6] Large-area graphene is set 5 µm away from the stimulating electrode and with parameters: $C = 0.02\text{F m}^{-2}$and $i_0 = 0.05\text{ A m}^{-2}$. The capacitance and exchange current density for graphene are estimated from the absorption measurement in supplementary figure 2a and leak current measurement in supplementary figure 2b, respectively. At t=0, both graphene and stimulating electrode are set at 0 V, after which a 200 ms pulse with 10 mV magnitude is applied to the stimulating electrode to simulate the experiment described in Figure 4c of the main text. The cross-section of simulation geometry is shown in supplementary figure 4a. The color map in supplementary figure 4a shows the simulated spatial distribution of electrolyte potential at t = 30 ms during the voltage pulse applied.

Supplementary figure 4b shows the voltage dynamics in solution just above graphene for three positions corresponding to A1 (below the tip), A2(37.5µm away) and A3(75µm away). The simulated local potential dynamics can be compared with the data

observed in Fig. 4c and Fig. 4d of the main text and provides a qualitative description for the spatio-temporal phenomenon observed in experiment.

The potential difference with graphene (solid lines) and without graphene (dashed lines) are shown in supplementary figure 4b. The potential difference is within 10%, indicating that the solution and probe are essentially blind to the presence of graphene, and that our detection schema may be treated as a truly nonperturbative readout of local electric fields.

The dynamics of the electric potential in solution can also be qualitatively described by the equivalent circuit shown in supplementary figure 4c. This equivalent circuit consists of the solution resistance, impedance from the electrode/solution interface, and impedance from the graphene/solution interface. The results of the calculation using the equivalent circuit are shown as dashed lines in Figure 4c.

Supplementary Note 5: Spatial resolution of CAGE imaging scheme

To demonstrate the spatial resolution of our CAGE imaging system, we spread polystyrene microspheres (with a 1 μm diameter) on the waveguide surface and analyzed the images formed by a single microsphere. Figure S5a shows an image of one microsphere taken with the CAGE optical system. The objective we use in the study is 10X MPlan objective with N.A. = 0.26. The resulting lower limit for spatial resolution is 7 μm, which is larger than the diameter of a polystyrene microsphere, therefore the microsphere can be treated as a point source and the intensity profile reports the point spread function of the optical system.

Supplementary figure 5b shows a intensity profile on a line cut across the direction perpendicular to the light propagation in the waveguide, $\vec{k}$. The full width at half maximum(FWHM) is measured to be 10μm, which is reflective of the spatial resolution in this direction. Supplementary figure 5c shows a intensity profile on a line cut across the direction parallel to $\vec{k}$. We observe a long tail of ~40 μm in the $\vec{k}_{||}$ direction. This is due to the slow decay of light over the waveguide propagation distance. The reflection coefficient of the waveguide depends sensitively on the incident angle. As the focused beam has a finite angular spread due to the uncertainty principle, interference is observed as the multiple secondary peaks observed in the $\vec{k}_{||}$ direction. With graphene as absorber and our designed waveguide structure, we estimate that the light decays in the tens of microns length scale which corroborates with the experimental observation. Despite the decay tail, the spatial resolution in the propagation direction determined by the FWHM is 16 μm.

Supplementary References

1. Zhu, S., Yu, A. W., Hawley, D. & Roy, R. Frustrated total internal reflection: A demonstration and review. *Am. J. Phys.* **54,** 601–607 (1986).
2. Jackson, J.D., *Classical electrodynamics 3rd edition* 385 (Wiley India Pvt. Limited, 2007).
3. Wang, F. *et al.* Gate-Variable Optical Transitions in Graphene. *Science* **320,** 206–209 (2008).
5. Horng, J. *et al.* Drude conductivity of Dirac fermions in graphene. *Phys Rev B* **83,** 165113 (2011).
6. Haynes, W. M. & Lide, D. R. *CRC handbook of chemistry and physics : a ready-reference book of chemical and physical data* D-221 (CRC Press, 2011).
7. Einevoll, G. T., Kayser, C., Logothetis, N. K. & Panzeri, S. Modelling and analysis of local field potentials for studying the function of cortical circuits. *Nat Rev Neurosci* **14,** 770–785 (2013).
